# Psychosocial factors related to physical activity in frail and prefrail elderly people

**DOI:** 10.1186/s12877-022-03042-2

**Published:** 2022-05-09

**Authors:** Fabio Jiménez-Zazo, David Navarrete-Villanueva, Alba Gómez-Cabello, Cristina Romero-Blanco, Esther Cabanillas, Jorge Pérez-Gómez, Sergio Calonge-Pascual, Ignacio Ara, Germán Vicente-Rodríguez, Susana Aznar

**Affiliations:** 1 PAFS Research Group, Faculty of Sports Sciencies, University of Castilla-La Mancha, 45071 Toledo, Spain; 2 Exercise and Health in Special Population Spanish Research Net (EXERNET), 50009 Zaragoza, Spain; 3 GENUD (Growth, Exercise, Nutrition and Development) Research Group, University of Zaragoza, 50009 Zaragoza, Spain; 4 Agrifood Research and Technology Centre of Aragón -IA2-, CITA-Universidad de Zaragoza, 50009 Zaragoza, Spain; 5 Faculty of Health, Department of Physiatry and Nursing, University of Zaragoza, 50009 Zaragoza, Spain; 6 Faculty of Health and Sport Science FCSD, Department of Physiatry and Nursing, University of Zaragoza, 50009 Zaragoza, Spain; 7 Defense University Center, 50090 Zaragoza, Spain; 8 Biomedical Research Net in Physiopatology, Obesity and Nutricition (CIBERObn), 28029 Madrid, Spain; 9 PAFS Research Group, Faculty of Nursing, University of Castilla-La Mancha, 13071 Ciudad Real, Spain; 10 HEME Research Group, Faculty of Sports Sciencies, 10003 Cáceres, Spain; 11 ImFINE Research Group, Department of Health and Human Performance, Faculty of Physical Activity and Sport Sciences-INEF, Polytechnic University of Madrid, 28040 Madrid, Spain; 12 GENUD Toledo Research Group, Universidad de Castilla-La Mancha, 45071 Toledo, Spain; 13 Biomedical Research Networking Center On Frailty and Healthy Aging (CIBERFES), 28029 Madrid, Spain

**Keywords:** Transtheoretical model, Older adults, Physical activity, Frailty

## Abstract

**Background:**

Increased physical activity (PA) is a very important factor in a healthy aging lifestyle. Psychosocial factors have also a main role in the initiation and maintenance of this behavior, but nowadays its implications for frailty elderly people are unknown, therefore, the aim of this study was to identify the psychosociological variables of behavior change that influence the practice of (PA) in frail and prefrail elderly.

**Methods:**

A total of 103 frail and pre-frail elderly people (72 females) participated in this cross-sectional study, on the framework of the EXERNET-Elder3.0 project. Age ranged from 68–94 years (mean = 80.4 ± 5.9 years). Individualized face-to-face interviews according to the constructs of the Transtheoretical Model of Change (TTM) [(decisional balance (DB) and self-efficacy (SE)], social support (SS) (family and friends) and outcome expectations (OE) were administered to all participants.

**Results:**

Significant differences were found in DB, perceived benefits (PBn), SE, family-related SS and OE as a function of stages of change (SoC) (*p* < 0.005), but no significant were found in perceived barriers (PBrr) (*p* = 0.259) and friends-related SS (*p* = 0.068). According to the Scheffé post-hoc test, those in advanced SoC (Action-Maintenance), scored higher than those in lower SoC (Precontemplation-Contemplation and Preparation).

**Conclusion:**

The scores obtained from the study variables differed according to the SoC, supporting the external validity for the use of the TTM in frailty elderly. Further research is needed to determine the impact of PBrr and friends-related SS on this people, as well as to identify the validity of this model in the long-term in this population.

## Background

Frailty is characterized by a progressive age-related decline in physiological systems that results in decreased reserves of intrinsic capacity, which confers extreme vulnerability to stressors and increases the risk of a range of adverse health outcomes [1]. Frailty is associated with aging, but it is not dependent on it, since frailty can be prevented and even reversed, delaying its appearance and decreasing vulnerability status [2].

Physical activity (PA) is considered one of the most effective strategies to promote health and one of the most important behaviors in a healthy aging lifestyle [3].

The inclusion of PA within older people’s lifestyles implies a change in behavior, where psychosocial determinants play an important role in the initiation and maintenance of PA pattern [4].

Behavioral theories help us to create a theorical framework to understand and predict behavior change in population. Behavior change is a complex process in which different mediators can act at the same time [5], so it is interesting to measure different constructs to identify behavior change more successfully.

The Transtheoretical Model of Change (TTM) proposed by Prochaska and DiClemente [6] assumes that behavior change is a dynamic process, which occurs through a temporal dimension in a sequence of stages and processes, by which the individual moves until reaching regular behavior. The TTM has reported a positive impacts on PA behavior [7], this model is composed mainly by four constructs: stages of change (SoC), processes of change, decisional balance (DB) and self-efficacy (SE). The SoC, evaluate the motivation to change and the current behavior change, is composed of 5 stages: Precontemplation, contemplation, preparation, action and maintenance. These stages classify individuals into groups ranging from unintentional change (precontemplation) to the acquisition of a habit after 6 months (maintenance). The DB, determine the perception of benefits (PBn) and barriers (PBrr) that the people evaluates to make a change. Self-efficacy determines the people's ability to act in a given way in a given situation being also considered one of the predictors of PA in elderly people [8], and finally, the processes of change, which are the tools (cognitive and behavioral) that the people uses to carry out the change.

Furthermore, other psychosocial determinants highlighted for PA promotion in elderly are social support (SS), offered by family and friends, and the outcome expectations (OE), understood as what the people expects to achieve with the proposed behavior change [4]. However, limited information exists on those determinants in this specific population.

It is important to explore and understand the mediators that influence PA-related behavioral change in frailty elderly people in order to improve PA promotion interventions tailored to their characteristics, aimed at encouraging the initiation and maintenance of an active long-term process of being active. To date, it is important to point out that many of the behavior change studies have been carried out in healthy elderly people, but not in frailty elderly people. These studies do not represent either older adults with illnesses and/or disabilities that could present different determinants in the promotion of PA [4]. In this sense.

Therefore, the aims of this study were to identify the psychosociological variables of behavior change that influence the practice of PA in frail and pre-frail elderly people and to analyze the relationship among them.

## Material and methods

### Study design and participants

This cross-sectional study was carried out in 2019 on the framework of the EXERNET-Elder3.0 project, whose complete methodology has been published previously [9]. Participants were recruited from four health care centers and three nursing homes from the city of Zaragoza, Aragón, Spain. These centers carried out an initial screening of all participants with the Clinical frailty scale (short adapted version) and Frail index test. From a total of 169 people initially selected, only 117 completed a face-to-face interview about psychosociological variables of behavior change and were tested with the Short Physical Performance Battery (SPPB) to allocate them in their state of frailty (frail and pre-frail). Inclusion criteria were: people aged ≥ 65 years with frail or pre-frail according to the SPPB (scores between 4 and 9 included). Exclusion criteria were: suffering cancer, dementia, or contraindications for exercise (indicated by the doctor). Finally, a sample of 103 elders (30,1% male and 69,9% female) met the inclusion criteria and were included in the present study. Written informed consent was obtained from all participants. The study has been approved by the Hospital Universitario Fundación de Alcorcón (16/50) Ethics Committee in Alcorcon, municipality of Madrid and was performed in accordance with the Helsinki Declaration (1961) revised in Fortaleza (2013).

### Socio-demographic characteristics

Personal information, such as age, gender, marital status, living arrangement, education status and economic status were collected through a structured EXERNET questionnaire.

### Frailty measurement

The initial screening was done through the application of two tools, the short adapted version of the Clinical frailty scale [10], which is a short assessment based on clinical judgment according with the degree of disease and dependence for daily living activities and the FRAIL index [11], composed of 5 items that encompass questions about fatigue, resistance, ambulance, illness and loss of weight. Subsequently, if the person was included into the frailty group, the level of frailty was assessed through the SPPB battery [12], composed of 3 tests: standing balance, gait speed and standing up test from a chair. Each test of this battery gives a score from 0 (worst performance) to 4 (best performance) points. The final score of the SPPB battery is the sum of the points of each of the tests, the final value ranges from 0 to 12 points. According to the score obtained we can classify the patient in: Person with severe limitation or disability (0–4 points), moderate or fragile limitation (4–6 points), mild or pre-fragile limitation (7–9 points) and minimal or robust limitation (10–12 points) [12]. In our study we selected whose older adults with scores between 4 and 9 points, as previous studies in frail and pre-frail population [13–15].

### Psychosocial Interviews

All psychosociological variables were measured through semi-structured individual interviews performed face-to-face. Each interview lasted approximately 20–30 min. Regular PA or exercise was described as those activities involving brisk walking, running, biking, swimming or any other activities where the exertion was at least as intense as these activities, at a frequency of at least 30 min/day or more, at least 5 days/week.

The SoC were measured through the Physical Activity Stage of Change Questionnaire [16]. This tool is composed of 4 dichotomous scale (yes/no) questions related to regular PA and intentions. This questionnaire allows us to categorize individuals into 5 stages: precontemplation (inactive, does not think about being more active), contemplation (inactive, but thinks about being more active), preparation (does some PA but is not regular), action (performs PA but for a period of less than 6 months) and maintenance (transform the practice of PA into a habit).

The DB was assessed through the Decisional Balance Scale for physical exercise [17]. The scale consists of 16 items, 10 that reflect the pros or benefits and 6 that states cons or barriers of PA. The answers were collected using a likert scale from 1 (not important) to 5 (very important), according to how they felt at that time.

The personal confident to exercise in different situations was measured through the Self-efficacy Questionnaire [18]. This questionnaire is composed of 5 items to be rated on a five-point likert scale from 1 (not at all confident) to 5 (extremely confident).

Family and friend SS for PA was assessed through the Social Support and Exercise Scale [19]. The questionnaire is composed of 13 items and determines the frequency of SS with a 5 point likert scale, ranging from 1 (None) to 5 (Very often).

The Outcome Expectations for Exercise Scale [20] values the expected benefits of the participant as a result of being physically active. The scale is composed of 9 items and evaluates the expected mental benefits through a likert scale with a range from 1 (disagree) to 5 (agree).

### Statistical Analysis

The results of the study have been analyzed using SPSS v.25. Descriptive statistics (reported as number of participants and percentage, according to the nature of these variables) and chi-square test were used to summarize and compare the psychosocial variables of behavior change regarding to the SoC. No significant differences were found between the frailty levels (frail and pre-frail) for the study variables. Therefore, the whole sample was used for the analyses. SoC were group into three groups: inactive stages (Precontemplation and Contemplation stages (PC)), non-regular active stage (Preparation stage (Pp)) and active stages (Action and Maintenance stages (AM)). The same groupings were made by Wilson et al., (2016) [21] in elderly with pathology. Tests for normality were performed by implementing Kolmogorov–Smirnov tests and parametric statistical (*p* > 0,05) were applied. ANOVA tests were used to determine significant differences between the psychosocial variables of behavior change in each grouped SoC and Scheffé post-hoc test was applied to determinate differences between groups. Finally, a Pearson correlation was performed to determine the relationship between all psychosocial variables of behavior change. The level of significance was set at *p* < 0.05.

## Results

The sample comprised of a total of 103 participants (72 females; 69.9%) ranging from 68 to 94 years (M = 80.40; SD = 5.91 years). According to the individual SoC, half of the participants were in maintenance stage (*n* = 50; 48.5%), followed by preparation stage (*n* = 18; 17.5%), action stage (*n* = 16; 15.5%), contemplation stage (*n* = 12; 11.7%) and precontemplation (*n* = 7; 6.8%). Using the grouped approach, participants were divided into PC stage (*n* = 19; 18.4%), Pp stage (*n* = 18; 17.5%) and AM stage (*n* = 66; 64.1%). The specific characteristics of the participants are shown in Table 1.

**Table 1:** Characteristics of the participants

	**Total Sample** (*n* = 103, 100%)	**PC** (*n* = 19, 18.4%)	**Pp** (*n* = 18, 17.5%)	**AM** (*n* = 66, 64.1%)	**p**
**Social-demographic**					
**Age** (years, Mean ± SD)	80.40 ± 5.9	79.16 ± 5.6	80.28 ± 6.4	80.79 ± 5.9	0.287
**Gender** (n,%)					
Male	31 (30.1%)	4 (21.1%)	5 (27.8%)	22 (33.3%)	0.573
Female	72 (69.9%)	15 (78.9%)	13 (72.2%)	44 (66.7%)	
**Marital status** (n,%)					
Married	14 (14%)	2 (10.5%)	3 (16.7%)	9 (13.6%)	0.075
Single	43 (43%)	12 (63.2%)	5 (27.8%)	26 (39.4%)	
Divorced	4 (4%)	2 (10.5%)	1 (5.6%)	1 (1.5%)	
Widower	39 (39%)	2 (10.5%)	9 (50.0%)	28 (42.4%)	
Missing	3 (2.9%)	1 (5.3%)	-	2 (3%)	
**Living arrangement** (n,%)					
Live alone	45 (45%)	10 (52.6%)	7 (38.9%)	28 (42.4%)	0.571
Missing	3 (2.9%)	1 (5.3%)	-	2 (3%)	
**Education status** (n,%)					
Do not know to read and to write	3 (3%)	-	1 (5.6%)	2 (3%)	0.774
Read and write, but no studies	15 (15%)	2 (10.5%)	3 (16.7%)	10 (15.2%)	
Primary studies	64 (64%)	12 (63.2%)	11 (61.1%)	41 (62.1%)	
Secondary studies	14 (14%)	4 (21.1%)	3 (16.7%)	7 (10.6%)	
University studies	4 (4%)	-	-	4 (6.1%)	
Missing	3 (2.9%)	1 (5.3%)	-	2 (3%)	
**Economic status** (n,%)					
Own income	74 (75.5%)	10 (52.6%)	15 (83.3%)	49 (74.2%)	0.819
Missing	5 (4.9%)	2 (10.5%)	-	3 (4.5%)	
**Frailty status** (n,%)					
Frail	27 (26.2%)	3 (15.8%)	7 (38.9%)	17 (25.8%)	0.277
Pre-frail	76 (73.8%)	16 (84.2%)	11 (61.1%)	49 (74.2%)	

*M* Mean, *SD* Standard Deviation, *PC* Precontemplation & Contemplation stages, *Pp* Preparation stage, *AM* Action & Maintenance stages

Statistical significance: *p* < 0.05

Table 1 show no significant differences were found in the participants characteristics by the SoC. Table 2 shows the means and standard deviations of each of the psychosocial variables of behavior change analyzed, across the classification in the 3 categories of the SoC. It can be observed how as we moved up thorough the SoC there was an increase in the values of SE and OE. In relation to DB and PBn, both increased from earlies to most advanced stages, highlighting the PC stages with the highest PBrr. According to the SS of family and friends, the highest results were observed in the most advanced SoC.

**Table 2:** Differences in psychosocial variables of behavior change across the stages of Change

	**PC**		**Pp**		**AM**		**p**	**Scheffé** **Post-Hoc**
	**n**	**M ± SD**	**n**	**M ± SD**	**n**	**M ± SD**		
**Self-efficacy**	19	2.45 ± 0.45	18	2.86 ± 0.46	66	3.64 ± 0.69	0.0001*	PC < AM, Pp < AM
**Decisional balance**	19	0.82 ± 0.76	18	0.93 ± 0.49	66	1.32 ± 0.53	0.001**	PC < AM, Pp < AM
**Benefits perceived**	19	3.87 ± 0.42	18	3.91 ± 0.34	66	4.17 ± 0.32	0.0001*	PC < AM, Pp < AM
**Barriers perceived**	19	3.04 ± 0.51	18	2.98 ± 0.46	66	2.85 ± 0.49	0.259	
**Outcome expectations**	19	3.78 ± 0.52	18	3.98 ± 0.71	66	4.44 ± 0.40	0.0001*	PC < AM, Pp < AM
**Family Social Support**	18	30.22 ± 6.39	18	29.72 ± 7.32	63	36.67 ± 7.78	0.0001*	PC < AM, Pp < AM
**Friends Social Support**	12	26.50 ± 5.35	14	24.86 ± 3.98	39	30.49 ± 9.96	0.068	

*M* Mean, *SD* Standard Deviation, *PC* Precontemplation & Contemplation stages, *Pp* Preparation stage, *AM* Action & Maintenance stages

Statistical significance: *p* < 0.05; * Statistical significance *p* < 0.001; ** Statistical significance *p* < 0.01

Among the psychosocial variables of behavior change, ANOVA found PBrr (*p* = 0.259) and friends SS (*p* = 0.068) not to be significantly different among the SoC. According to the Scheffé post-hoc test, those in the AM stages had higher SE, DB score, more PBn, higher OE and more family-related SS that those in lower stages (Table 2).

The statistically significant Pearson correlation coefficients between psychosocial variables of behavior change are shown in Table 3. Most of the psychosociological variables were significantly related to each other. Only those which value is greater than 0,4 will be outlined. The SoC were significantly and positively associated with the variables of SE, PBn and OE, determining that, more advanced SoC were related to higher SE, PBn and OE. Similarly, the SE score showed a significant positive relationship with the OE, DB score and PBn. The PBn showed a significant positive association with the OE and family SS. Finally, the OE showed a significant positive relationship with family SS. The variables of PBn and PBrr showed a strong significant positive and negative association respectively with the DB score, but this is due to they are part of the same construct.

**Table 3:** Correlation between psychosocial variables of behavior change

	**1**	**2**	**3**	**4**	**5**	**6**	**7**	**8**
1. Stages of change	-	**.649**^a^	.347^a^	**.410**^a^	-.145	**.495**^a^	.390^a^	.318^a^
2. Self-efficacy		-	**.482**^a^	**.424**^a^	-.298^a^	**.564**^a^	.247^b^	.093
3. Decisional balance			-	**.590**^a^	**-.776**^a^	.335^a^	.233^b^	.191
4. Benefits perceived				-	.008	**.425**^a^	**.429**^a^	.166
5. Barriers perceived					-	-.073	.067	.091
6. Outcome expectations						-	**.462**^a^	.248^b^
7. Family social support							-	.296^b^
8. Friends social support								-

^a^The correlation is significant at the 0.01 level (bilateral)

^b^The correlation is significant at the 0.05 level (bilateral)

## Discussion

The purpose of this study was to evaluate different psychosociological variables of behavior change related to PA in frail and prefrail elderly over 65 years. More specifically, it was hypothesized that advanced SoC (action or maintenance) for PA would be related to greater scores in SE, DB, PBn, OE, family and friend SS and lower scores in PBrr, than those in lower stages.

Our study is the first which has analyzed the SoC for PA in frail and pre-frail elderly over 65 years. The results showed that DB score increased across to the SoC. The same trend has been observed in other studies with elderly [22–24]. Moreover, the participants in AM stages, perceived significantly more benefits to be physically active than those in PC and Pp stages, these results support previous research in elderly people in which at initial SoC perceived lower benefits compared to more advanced stages [22–25]. However, the literature in healthy elderly has also revealed some contrary results where they did not find significant differences between the PBn according to the SoC [21, 26]. On the other hand, our study has shown a decrease in the PBrr to PA score in the advanced SoC (AM) as already seen in the literature of healthy elderly people [21, 22, 24, 26]. The study of Kosma & Cardinal (2016) [26] showed that self-PBrr contributed to explain only 11% of the variance in SoC. However, in our data, no significant differences between PBrr and SoC were found. Similar results than us were found in other studies [22, 25]. This outcome could be due to the fact that PBrr to PA in frail older people could be specific and differ from those already outlined in healthy elderly. Therefore, it seems necessary to research in depth PBrr to PA in frail and prefrail elderly people. This need was revealed by Ellis et al., (2007) [27] when they studied this same association in elderly people with physical disabilities. Finally, we found a significant relationship between the PBn to be physically active with the OE and the SoC which confirms the SoC appear to be applicable and adequate for frailty elderly population.

In relation to SE, we found significant differences among SE score at the different SoC, observing an incremental trend as we progress in the SoC, which corroborates the data supported by the scientific literature for healthy elderly [22–26, 28]. The same incremental trend was observed but without finding significant differences in other study [21].Our data supports a direct significant relationship among grouped SoC with SE score, OE, DB score and PBn, suggesting that as the ratings on these variables increase, a progress towards a more active SoC and vice-versa can be observed. Different studies in elderly people have determined SE as one of the predictors of SoC [24, 26], practice of PA [22, 23, 26, 28] and the risk of falls [26], however, our results provide novel evidences in frail and pre-frail elderly, suggesting that psychological determinants are important also in vulnerable elders to get enroll in PA or exercise programs.

Social support is one of the determining factors in the initiation and maintenance of PA in older people [4]. Within our study, we have been able to identify that the most advanced SoC were significantly associated with higher family and friends-related SS scores compared to the initial stages. Studies have shown how SS influences SoC [29]. In our study, family SS significantly increased from early SoC to later ones. Recent studies show that a greater self-perception of family SS is related to higher PA levels and meeting PA guidelines [30]. Moreover, there is also evidence that suggests that friends SS is inversely associated with frailty [31]. However, no significant differences were found for friends-related SS and SoC for PA in our study. We need to take into account, that frail elderly people may have a reduced group of friends, which may not be accessible for them all the time. Hence, some research in healthy elderly people has supported the same non relationship [22].

As hypothesized, our results showed a significant progressive increase in the OE score according to the SoC. This provides evidence of the OE plays a very important role in the initiation and maintenance of PA in the frail elderly group, as it has already been manifested in healthy elderly people [4].

Our findings support the use of the TTM and SoC for PA in frail and pre-frail elderly people. In fact, the advanced SoC (action or maintenance) obtain better results in each psychosociological variable of behavior change with respect to those participants at initial SoC (precontemplation and contemplation) confirming that the structure of the behaviour change process appears to be the same as in other populations. Frail and prefrail individuals move from being unaware or unwilling to practice PA to considering the possibility of change (i.e. be physically active), then to becoming determined and prepared to make the change, and finally to taking action and sustaining or maintaining that change over time (i.e. adopt an active lifestyle).

Currently, the scientific literature on PA-related behavior change in frailty elderly is scarce. This study expands the knowledge in this science field, identifying the application of psychosocial variables of behavior change across grouped SoC in frail and prefrail elderly. Placing people into a stage of change helps to improve understanding and predicting PA behavior. This information is important for researchers, health professionals and health promotors as it will be useful for the development of future tailored PA interventions, based in these variables of behavior change and, finally, to promote an increase of PA levels.

This study has several limitations. Firstly, it was a observational cross-sectional study in which the more than half of participants were in action or maintenance stages (64.1%), a grouped stages approach was used in the statistical analysis, due to the low representativeness of individuals in the pre-contemplation and contemplation stages, the same groupings were made in elderly with pathology [21]. Secondly, the sample size was small and it was composed of institutionalized and non-institutionalized frailty elderly. Finally, no relationship was found between PBrr and SoC, this may indicate that this population presents specific barriers that limit their practice of PA, independently of the PBrr by healthy elderly people or those with other pathologies, therefore, future researches are needed whose have more equitable samples, distributed in a similar way between each of the SoC and longitudinal designs to study these constructs in a more specific way, using scales with greater sensitivity that help to identify more effectively the possible perceived influences by frailty elderly people and that limit their practice of PA.

## Conclusion

This study examined the SoC for PA behavior among elderly with frailty (frail and pre-frail) according to the constructs of TTM (DB and SE), SS (family and friends) and OE. The scores obtained from these variables differed according to the SoC, supporting the external validity use with frailty elderly people. The results obtained from this study will allow the creation and development of future tailored PA interventions and multidisciplinary programs focused on this group of patients. Further research is needed to identify to validate this model in long-term behavior change with frailty elderly people.

## Data Availability

The datasets used and/or analysed during the current study are available from the corresponding author on reasonable request.

## Abbreviations

PA: Physical Activity
TTM: Transtheoretical Model Of Change
SoC: Stages of Change
PC: Precontemplation and Contemplation Stages
Pp: Preparation Stage
AM: Action and Maintenance Stages
DB: Decisional Balance
PBn: Perceived Benefits
PBrr: Perceived Barriers
SE: Self-Efficacy
SS: Social Support
OE: Outcome Expectations

## References

[1] World Health Organization. World report on ageing and health: World Health Organization; 2015 [Available from: https://apps.who.int/iris/bitstream/handle/10665/186466/9789240694873_spa.pdf?sequence=1.

[2] Ahmed N, Mandel R, Fain MJ (2007). Frailty: an emerging geriatric syndrome. Am J Med.

[3] Cartee GD, Hepple RT, Bamman MM, Zierath JR (2016). Exercise Promotes Healthy Aging of Skeletal Muscle. Cell Metab.

[4] Stralen M, de Vries H, Mudde A, Bolman C, Lechner L (2009). Determinants of initiation and maintenance of physical activity among older adults: A literature review. Health Psychol Rev.

[5] Baranowski T, Anderson C, Carmack C (1998). Mediating variable framework in physical activity interventions. How are we doing? How might we do better?. Am J Prev Med.

[6] Prochaska JO, DiClemente CC (1983). Stages and processes of self-change of smoking: toward an integrative model of change. J Consult Clin Psychol.

[7] Gourlan M, Bernard P, Bortolon C, Romain AJ, Lareyre O, Carayol M (2016). Efficacy of theory-based interventions to promote physical activity. A meta-analysis of randomised controlled trials. Health Psychol Rev.

[8] French DP, Olander EK, Chisholm A, Mc SJ (2014). Which behaviour change techniques are most effective at increasing older adults' self-efficacy and physical activity behaviour?. A systematic review Ann Behav Med.

[9] Fernández-García ÁI, Gómez-Cabello A, Moradell A, Navarrete-Villanueva D, Pérez-Gómez J, Ara I (2020). How to Improve the Functional Capacity of Frail and Pre-Frail Elderly People? Health, Nutritional Status and Exercise Intervention. The EXERNET-Elder 3.0 Project. Sustainability.

[10] Rockwood K, Song X, MacKnight C, Bergman H, Hogan DB, McDowell I (2005). A global clinical measure of fitness and frailty in elderly people. CMAJ.

[11] Morley JE, Malmstrom TK, Miller DK (2012). A simple frailty questionnaire (FRAIL) predicts outcomes in middle aged African Americans. J Nutr Health Aging.

[12] Guralnik JM, Simonsick EM, Ferrucci L, Glynn RJ, Berkman LF, Blazer DG (1994). A short physical performance battery assessing lower extremity function: association with self-reported disability and prediction of mortality and nursing home admission. J Gerontol.

[13] Verlaan S, Aspray TJ, Bauer JM, Cederholm T, Hemsworth J, Hill TR (2017). Nutritional status, body composition, and quality of life in community-dwelling sarcopenic and non-sarcopenic older adults: A case-control study. Clin Nutr.

[14] Bauer JM, Verlaan S, Bautmans I, Brandt K, Donini LM, Maggio M (2015). Effects of a vitamin D and leucine-enriched whey protein nutritional supplement on measures of sarcopenia in older adults, the PROVIDE study: a randomized, double-blind, placebo-controlled trial. J Am Med Dir Assoc.

[15] Courtwright AM, Zaleski D, Tevald M, Adler J, Singer JP, Cantu EE (2019). Discharge frailty following lung transplantation. Clin Transplant.

[16] Marcus BH, Rossi JS, Selby VC, Niaura RS, Abrams DB (1992). The stages and processes of exercise adoption and maintenance in a worksite sample. Health Psychol.

[17] Marcus BH, Rakowski W, Rossi JS (1992). Assessing motivational readiness and decision making for exercise. Health Psychol.

[18] Marcus BH, Selby VC, Niaura RS, Rossi JS (1992). Self-efficacy and the stages of exercise behavior change. Res Q Exerc Sport.

[19] Sallis JF, Grossman RM, Pinski RB, Patterson TL, Nader PR (1987). The development of scales to measure social support for diet and exercise behaviors. Prev Med.

[20] Resnick B, Zimmerman SI, Orwig D, Furstenberg AL, Magaziner J (2000). Outcome expectations for exercise scale: utility and psychometrics. J Gerontol B Psychol Sci Soc Sci.

[21] Wilson JJ, Kirk A, Hayes K, Bradbury I, McDonough S, Tully MA (2016). Applying the Transtheoretical Model to Physical Activity Behavior in Individuals With Non-Cystic Fibrosis Bronchiectasis. Respir Care.

[22] Kim Y, Kosma M (2012). Psychosocial and Environmental Correlates of Physical Activity Among Korean Older Adults. Res Aging.

[23] Abbaspour S, Farmanbar R, Njafi F, Ghiasvand AM, Dehghankar L (2017). Decisional balance and self-efficacy of physical activity among the elderly in Rasht in 2013 based on the transtheoretical model. Electron Physician.

[24] Salehi L, Eftekhar H, Mohammad K, Taghdisi MH, Shojaeizadeh D (2010). Physical activity among a sample of Iranians aged over 60 years: an application of the transtheoretical model. Arch Iran Med.

[25] Kirk A, MacMillan F, Webster N (2010). Application of the Transtheoretical model to physical activity in older adults with Type 2 diabetes and/or cardiovascular disease. Psychol Sport Exerc.

[26] Kosma M, Cardinal BJ (2016). The Transtheoretical Model, Physical Activity, and Falls Risks Among Diverse Older Adults. Act Adapt Aging.

[27] Ellis R, Kosma M, Cardinal BJ, Bauer JJ, McCubbin JA (2007). Physical activity beliefs and behaviour of adults with physical disabilities. Disabil Rehabil.

[28] Guicciardi M, Lecis R, Anziani C, Corgiolu L, Porru A, Pusceddu M (2014). Type 2 diabetes mellitus, physical activity, exercise self-efficacy, and body satisfaction An application of the transtheoretical model in older adults. Health Psychol Behav Med.

[29] Resnick B, Nigg C (2003). Testing A Theoretical Model of Exercise Behavior for Older Adults. Nurs Res.

[30] Lindsay Smith G, Banting L, Eime R, O'Sullivan G, van Uffelen JGZ (2017). The association between social support and physical activity in older adults: a systematic review. Int J Behav Nutr Phys Act.

[31] Chon D, Lee Y, Kim J, Lee KE (2018). The Association between Frequency of Social Contact and Frailty in Older People: Korean Frailty and Aging Cohort Study (KFACS). J Korean Med Sci.

